# Carbon source dependent promoters in yeasts

**DOI:** 10.1186/1475-2859-13-5

**Published:** 2014-01-09

**Authors:** Katrin Weinhandl, Margit Winkler, Anton Glieder, Andrea Camattari

**Affiliations:** 1 Austrian Centre of Industrial Biotechnology, Graz, Austria; 2 Institute of Molecular Biotechnology, Technical University Graz, Graz, Austria

## Abstract

Budding yeasts are important expression hosts for the production of recombinant proteins.

The choice of the right promoter is a crucial point for efficient gene expression, as most regulations take place at the transcriptional level. A wide and constantly increasing range of inducible, derepressed and constitutive promoters have been applied for gene expression in yeasts in the past; their different behaviours were a reflection of the different needs of individual processes.

Within this review we summarize the majority of the large available set of carbon source dependent promoters for protein expression in yeasts, either induced or derepressed by the particular carbon source provided. We examined the most common derepressed promoters for *Saccharomyces cerevisiae* and other yeasts, and described carbon source inducible promoters and promoters induced by non-sugar carbon sources. A special focus is given to promoters that are activated as soon as glucose is depleted, since such promoters can be very effective and offer an uncomplicated and scalable cultivation procedure.

## Introduction

Recombinant protein production in yeast has represented, in the last thirty years, one of the most important tools of modern biotechnology. The possibility to express a high amount of a single protein, separated from its original context, allowed major leaps forward in the understanding of many cellular functions and enzymes. However, since every host has its specific genetic system, species-specific tools have been established for each individual host/vector combination. In particular, promoters drive the transcription of the gene of interest and therefore are key parts of efficient expression systems to produce recombinant proteins. Furthermore expression of enzyme cascades and whole heterologous or synthetic pathways fully relies on a tool box of promoters with different sequence and properties.

Typically, there are two major choices concerning transcription of a gene of interest: inducible or constitutive promoters. The decision for one of these alternatives depends on the specific requirements of a bioprocess and the properties of the target protein to be produced. Constitutive expression, performed by a range of very strong promoters like P_*GAP*_ (glycerinaldehyde-3-phosphate dehydrogenase) [1], P_*PGK1*_ (3-Phosphoglyceratekinase) [2] or P_*TEF1*_ (translation elongation factor) [3] from *Saccharomyces cerevisiae* is not always preferable, since recombinant proteins can have a toxic effect on their host organism at constantly high expression level.

Controllable gene expression can be achieved with inducible and derepressed promoters. Most of these inducible promoters are responsive to catabolite repression or react to other environmental conditions, such as stress, lack or accumulation of essential amino acids, ion concentrations inside the cell and others [4-6]. For practical applications, carbon source dependent promoters have the main advantage in the segregation of the host growth phase from the protein production phase, allowing maximizing growth before inducing a potentially burdening expression phase. Very recently, Da Silva & Srikrishnan have summarized important tools for controlled gene expression and metabolic engineering in *S. cerevisiae*, such as useful vectors, promoters and the procedure of chromosomal integration of recombinant genes [7].

In order to categorize a large amount of information, and due to its practical importance, in this review we describe the various promoters according to their basic behavior in relation to carbon sources. This includes the most essential regulatory elements and mechanisms of carbon source regulation as described by the main chapters of this review: glucose repression in yeast and promoters which are either induced by simple de-repression or induced by carbohydrates or other non sugar carbon sources.

**Figure 1:**
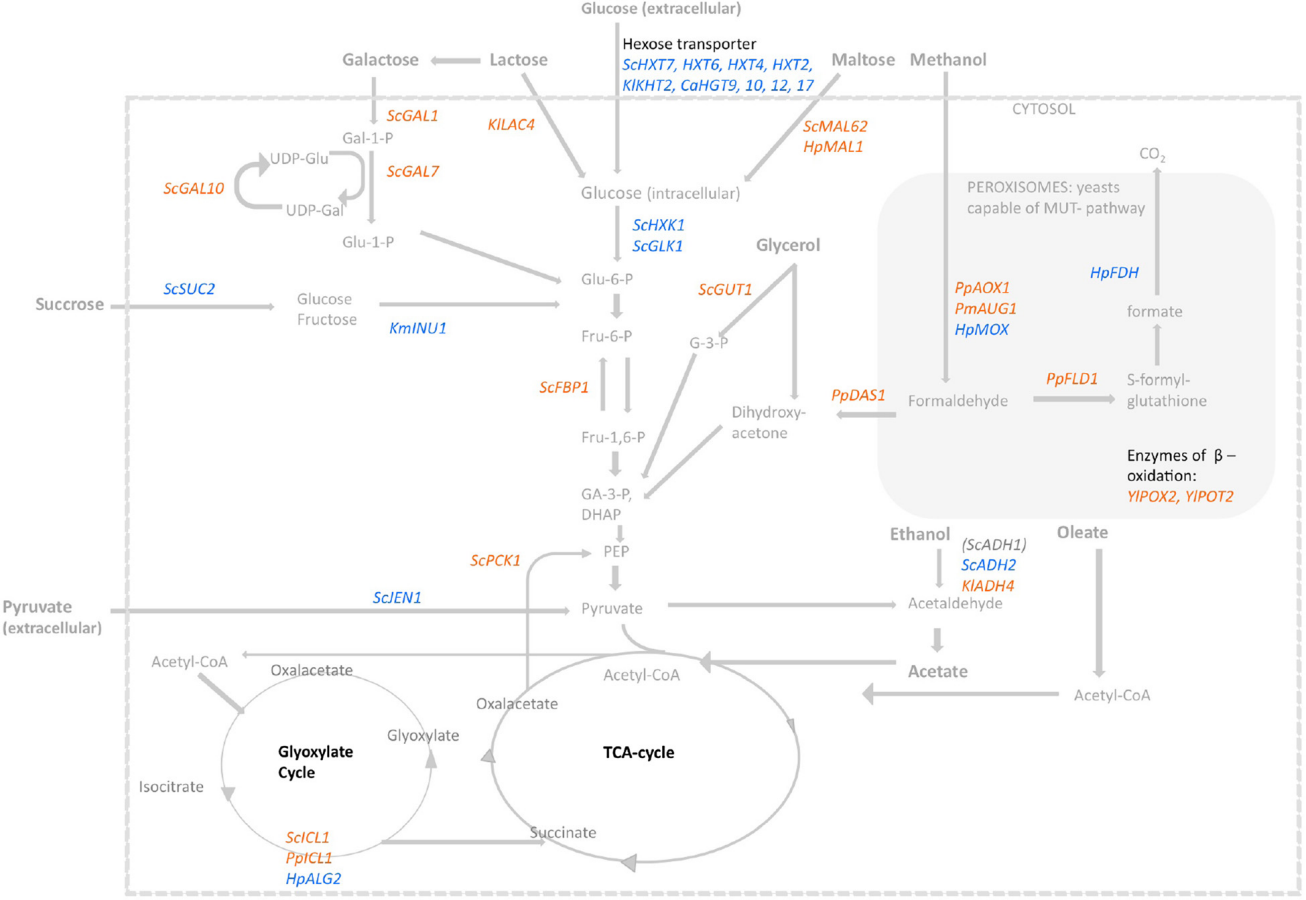
Target genes for inducible (orange) and derepressed (blue) carbon source dependent promoters in yeasts and their localization in the metabolism.

Wherever possible, special emphasis is given on the applicability of individual promoters in different hosts and application spectra for industrial protein synthesis. Figure 1 gives an overview of the particular target promoters described within this work and their localization in the yeast cell metabolism.

### Glucose repression in yeasts

Glucose is a favored carbon and energy source in yeast. Glucose repression and derepression essentially concern genes involved in oxidative metabolism and TCA (tricarboxylic acid) cycle, genes encoding for the metabolism of alternative carbon sources (e.g. sucrose, maltose, galactose), or genes for gluconeogenesis [8-10]. In presence of glucose, decrease in transcription or translation at the gene level or increase in protein degradation at the protein level are the most common mechanism to regulate the gene products involved [11].

In an early attempt to clarify carbon source dependence in *S. cerevisiae*, Gancedo has listed the elements of catabolite repression in yeast, focusing on regulatory elements on transcriptional level (Table 1), which was extended to additional proteins such as Oaf1 or Mig2 and Mig3.

**Table 1:** **Promoter interacting elements of catabolite repression in *Saccharomyces cerevisiae *** **(as reviewed in**[10], [11], [12])

**Element**	**Designation**	**Function**
Activator (DNA-binding proteins)	Hap2/3/4/5 complex	Activates transcription of proteins for respiratory functions
	Gal4	Activates transcription of proteins for galactose and melobiose metabolism
	Mal63	Activates transcription of proteins for maltose utilization
	Adr1, Cat8, Sip4	Activates transcription of proteins for ethanol, glycerol and lactate utilization, as well as for gluconeogenic proteins
	Oaf1	Activates transcription of proteins for oleate utilization
Repressor (DNA-binding proteins)	Mig1 (Mig2, Mig3)	Recruits Ssn6-Tup1 complex (repressor complex) in glucose repressed genes
Intermediate elements	Snf1	Protein kinase (in complex with Snf4); derepression of glucose-repressed genes by phosphorylation of Mig1
	Glc7	Protein phosphatase; dephosphorylation of Snf1
Glucose signaling	Hxt-proteins	Hexose transporter
	Snf3	Glucose transporter
	Rgt2	Glucose transporter
	Hxk-proteins	Hexokinase
		Phosphorylation of glucose

The current understanding of the mechanism of glucose derepression suggests that first of all the presence of glucose has to be signaled to the related genes. This signal transduction is likely performed by hexose transporters (*HXT*-gene products, Rgt2, Snf3) and hexokinases (*HXK* gene products). In yeast cells, a fully functional hexose transport is essential to provide functional glucose repression events, since repression is prompted by uptake and metabolism of glucose [13]. This is consistent with the phenotype of a *HXT* deletion strain [14], and also with the observation that the AMP/ATP ratio reflects the glucose level inside the cell (a high AMP/ATP ratio leads to activation of Snf1 [9], a kinase directly involved in gene regulation by carbon sources). However, most likely the processed metabolite of monosaccharides in the cell–glucose-6-phosphate–is the main signal that activates glucose repression [15].

**Figure 2:**
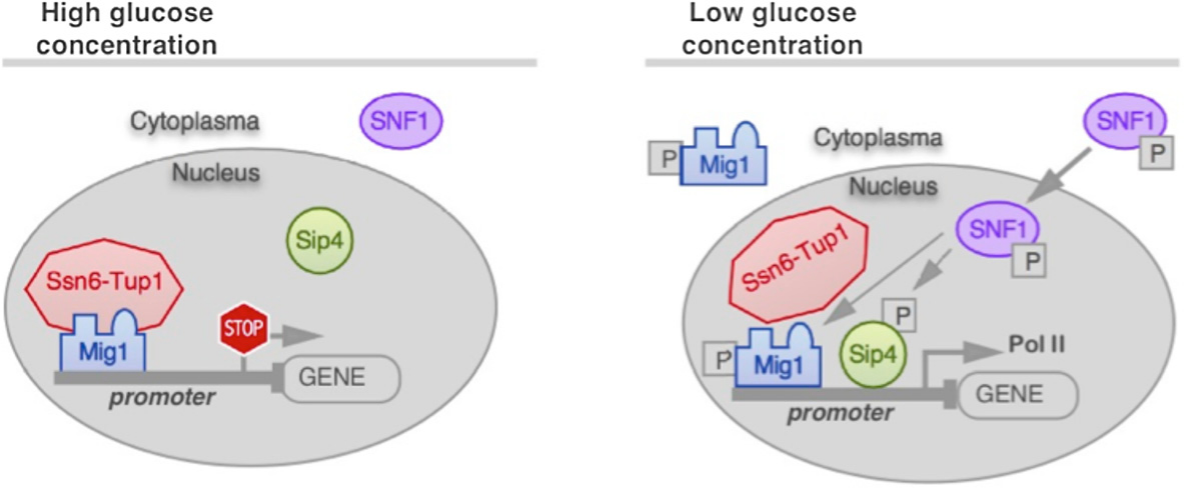
**Mechanism of glucose repression in yeast; modified from **[16]**.**

The event of glucose repression usually follows glucose level recognition, by repressors belonging to the Mig family comprising a group of C_2_H_2_-zinc-finger DNA-binding proteins. This family takes the name after Mig1, the most important repressor protein in this context, regulating the majority of glucose repressed genes (Figure 2).

At high glucose level, Mig1 is transferred from the cytoplasm into the nucleus, where it binds a GC-rich recognition site in the promoter sequence (for consensus sequences see Table 2), and recruits a repressor complex consisting of Ssn6-Tup1 [17-19]. Using *SUC2* promoter as a reporter system, it has been observed that the binding of Mig1 leads to a conformational change of the chromatin structure, further reinforced by Tup1 interaction with histones H3 and H4. Consequently, transcription initiating factors (such as Sip4) have no access to their binding sites [20].

**Table 2:** **DNA-motifs for regulator protein binding in natural promoter sequences of carbon source dependent *S. cerevisiae *** **promoters**

**DNA-binding protein**	**Consensus sequence**	**Reference**
Mig1	SYGGGG	[11]
Gal4	CGGASGACAGTCSTCCG	[11]
Mal63	GAAAWTTTCGC	[11]
Cat8	YCCNYTNRKCCG	[21]
Sip4	TCCATTSRTCCGR	[21]
Adr1	TTGGRG	[22]
Oaf1	CGGN_3_TNAN_9-12_CCG	[22]
Hap2	TNATTGGT	[22]

Many glucose repressed genes, for example hexose transporters (e.g. *MTH1*, *HXT4*, *HXK1*), are solely affected by Mig1-repression. However, two more Mig repressors (Mig2 and Mig3) are reported to be involved in glucose repression, by partly assisting Mig1 in a synergistic way (e.g. *ICL1*, *ICL2*, *GAL3*, *HXT2*, *MAL11*, *MAL31*, *MAL32*, *MAL33*, *MRK1*, *SUC2* are repressed by Mig1 and Mig2) or taking over complete repression events in some genes without the intervention of Mig1 activity (*SIR2* is repressed by Mig3). The involvement of a particular Mig repressor in gene expression is strongly correlated to glucose concentrations inside the cell, as has been observed for *HXT* genes [10].

Generally, *MIG1* from several yeast species are highly conserved, but there are some differences in regulation of homologous genes in different yeasts. One example is *GAL4* of *Saccharomyces cerevisiae*, which is regulated by Mig1 as described above, although *GAL4* homologue *LAC9* in *Kluyveromyces lactis* is triggered by a regulatory function of *KlGAL1* and has no Mig1 binding site [18].

As soon as glucose is depleted, the protein kinase Snf1 is activated, mediating the release of Mig1 and the repressor complex by phosphorylation. Subsequently, Mig1 is exported from the nucleus, the promoter is derepressed and the gene expression gets activated [8]. Again, in the *SUC2* expression model, the ATPase activity of the complex Swi/Snf triggers an ATP-dependent change of nucleosomal structure (chromatin remodeling) and facilitates the binding of transcription factors [20,23]. Consequently, activator proteins are binding to particular consensus sequences (Table 2) and initiate transcription [21,22,24].

### Promoters derepressed by carbon source depletion

The peculiarity of all these promoters (Table 3), all induced at low glucose levels, lays in the lack of a proper induction for their activity. Such a behavior, in fact, represents also a reason for interest in potential applications, as the expression of the protein of interest does not start during cell growth, when the carbon source is typically abundant, but only at the late exponential phase, allowing *de facto* a regulated gene expression without external induction step. The advantage of these promoters is even more promising moving from batch cultivations to fed-batch processes: during the feeding phase, a strict control on growth rate (and, in turns, on carbon source concentration in the fermenter) can be easily achieved, hence having a tight control on recombinant protein production with relatively simple fermentation procedures.

**Table 3:** Yeast promoters derepressed by gradual glucose consumption (repressed by glucose), and respective known regulator elements and binding sites

**Promoter**	**Protein function**	**Organism**	**Derepressed by: (strength)**	**Regulating sequence**	**DNA-binding target protein**	**Ref.**
HXT7	High affinity hexose transporter	*S. cerevisiae*	Low glucose level (10-15×)	No information available		[25][26]
HXT2	High affinity hexose transporter	*S. cerevisiae*	Low glucose level (10-15×)	-590 to -579	Rgt1	[27][28]
				-430 to -424		
				-393 to -387		
				-504 to -494	Mig1	[27]
				-427 to -415		
				-291 to -218	UAS	[29]
				-226 to -218	Activator protein?	[29]
HXT4	High affinity hexose transporter	*S. cerevisiae*	Low glucose level	-645 to -639	Rgt1	[28]
HXT6	High affinity hexose transporter	*S. cerevisiae*	Low glucose level (10×)	No information available	Mig2	[10]
KHT2	High affinity hexose transporter	*K. lactis*	Low glucose level (2×)	No information available		[30]
HGT9, 10, 12, 17	High affinity hexose transporter	*C. albicans*	Low glucose level	No information available		[31]
SUC2	Invertase	*S. cerevisiae*	Sucrose low glucose level (200×)	-499 to -480	Mig1/2	[20]
				-442 to -425		
				-627 to -617	Sko1	
				-650 to -418	UAS	
				-133	RNA-Pol II	
ADH2	Alcohol dehyrogenase	*S. cerevisiae*	Low glucose level (100×)	-319 to -292	Cat8	[24]
				-291 to ??	Adr1	
JEN1	Lactate permease	*S. cerevisiae*	Low glucose level (10×), lactate	-651 to -632	Cat8	[21][32]
				-1321 to -1302		
				-660 to -649	Mig1	[32]
				-1447 to -1436		
				-739 to -727	Abf1	[32]
MOX	Methanol oxidase	*H. polymorpha*	Low glucose level, glycerol	-245 to -112	Adr1	[33]
				-507 to -430	UAS	[34]
AOX delta 6	Alcohol oxidase	*P. pastoris*	Low glucose level, glycerol		deleted GCR1-site	[33]
GLK1	Glucokinase	*S. cerevisiae*	Low glucose level (6×), ethanol (25×)	-881 to -702	Gcr1	[35]
				-572 to -409	URS	
				-408 to -104	Msn2/4	
HXK1	Hexokinase	*S. cerevisiae*	Low glucose level (10×), ethanol	No information available		[36]
ALG2	Isocitrate lyase	*H. polymorpha*	Low glucose level	No information available		[37]

These promoter regions attract the binding of special transcription factors (e.g. Adr1), but as long as the carbon source is available, the chromatin structure is organized in such a way that the promoter is inaccessible to the activator protein. In the case of glucose, when its concentrations decreases, dephosphorylation of DNA-binding domains (as well as acetylation of histones H3 and H4) occurs, leading to a conformational change of the DNA region. Subsequently, the promoter region is accessible and gene expression can be activated by the activator protein without any induction signal [38].

Recently, Thierfelder and colleagues presented a new set of plasmids for *Saccharomyces cerevisiae*, containing several glucose dependent promoters induced at a low level of glucose (P_*HXK1*_, P_*YGR243*_, P_*HXT4*_, P_*HXT7*_; [39]). In *Pichia pastoris*, a set of 6 novel glucose dependent promoters was described; promoters of hexose transporters, of a mitochondrial aldehyde dehydrogenase and of some proteins with unknown function were represented in this list. Generally, all of them were also activated during glucose starvation [40].

### Hexose transporter genes in S. cerevisiae and other yeasts

Hexose transporters in *S. cerevisiae* are encoded by 17 *HXT* genes. Some of them are induced (e.g. *HXT1*), whereas others are repressed by high levels of glucose (e.g. *HXT2*, *HXT4*, *HXT7*) [41]. In this section we will focus on the glucose-repressed fraction of *HXT* genes, that includes all high-affinity glucose transporters. In addition, high-affinity hexose transporters from other yeasts, that may have the potential of good promoter activity, will be discussed.

Hexose transporter proteins Hxt2, 4, 6 and 7 in *S. cerevisiae* are repressed by high glucose concentration, and induced when glucose concentration decreases below a certain level [39]. Two independent transcription repression mechanisms apply, mediated respectively by Mig repressor (high glucose level) or by Rgt1, a C_6_-zinc cluster (no glucose). Both proteins are responsible for recruiting the Ssn6-Tup1 complex [29]. While derepression upon Mig1 release is dependent by Snf1, Rgt1 dissociation requires Grr1-mediated phosphorylation, which is dependent from Mth1 and Std1 activities [42]. Interestingly, another regulatory complex, depending on pH and the corresponding altered calcineurin pathway, was hypothesized. This assumption is based on observations on *HXT2* regulation: after shifting the media pH to 8, the expression of *HXT2* reaches a plateau, while in *snf1* mutant strains the expression was not completely inhibited. It was suggested that *HXT2* promoter might be a target for the transcription factor Crz1, which is active at high pH and activates the calcineurin pathway, a response to environmental stress in yeast. Also related to pH shift, although to a lesser extent, is the induction of *HXT7* and other glucose dependent proteins like Hxk1, Tps1, and Ald4. Overall, the response to alkaline stress of genes involved in glucose utilization suggests an impairment of glucose metabolism, probably due to a disturbed electrochemical gradient and subsequent uptake of nutrient through the cell wall: a sudden increase of pH value is a signal for the activation of stress responsive enzymes (e.g. superoxide dismutase, SOD) in order to maintain an appropriate pH for a functioning electrochemical gradient [27].

Many hexose transporter genes are not well described yet. Greatrix and colleagues compared the expression levels of *HXT1-17. HXT13*, for example, showed similar induction characteristics as *HXT2* (*i.e.* induction at 0.2% w/v glucose). Furthermore, *HXT6*, closely related to *HXT7*, is induced at low glucose concentrations [43], but its expression is more dependent on the Mig2 repressor [10].

*HXT7* seems to bind glucose with the highest affinity among all glucose transporters, and this fact is associated to a strong induction at low glucose level. The *HXT7* promoter region turned out to be suitable for recombinant protein production in yeast and was compared to other yeast promoters (P_TEF1_, P_ADH1_, P_TPI1_, P_PGK1_, P_TDH3_ and P_PYK1_) using lacZ as a reporter gene. Among them, P_HXT7_ was stated as the strongest promoter in continuous culture with limited glucose level [44]. Also in comparison with P_ADH1_ for *SUC2*- and *GFP*-expression, respectively, P_HXT7_ produced promising results [25].

A variant of P_HXT7_ (P_HXT7-391_, 5′ deletion [26]), showing strong constitutive expression, was applied for overexpression of phosphoglucomutase 2 to improve anaerobic galactose metabolism [45].

P_*HXT2*_ was successfully used for the recombinant production of squalene synthase (*ERG9*), which plays an important role in synthesis of compounds for perfumes and pharmaceuticals [46].

As expected, the characterization of hexose transporters, and relative promoters, is poorly characterized in less conventional yeasts. Nevertheless, *KHT1* and *KHT2* from *K. lactis*, *GHT1-6* from *Schizosaccharomyces pombe*, or *HGT*-genes from *C. albicans* have been described [47,48].

*KHT1* and *2* represent a sort of genetic anomaly, as both are located in a polymorphic gene locus of *RAG1*[30], which encodes either a low (Kht1, Rag1) or a moderate affinity hexose transporter (Kht2). Therefore, P_*KHT2*_ is more interesting for application where a more sensitive glucose dependent promoter element is required. *KHT2* turned out to be, sequence-wise, a close relative of *HXT7* and is similarly regulated. It has to be considered that *KHT2* is only weakly repressed by high glucose level and about 2-fold induced at concentrations below 0.1% (w/v) [49]. To our knowledge, the *KHT*2 promoter has not yet been applied for recombinant protein production so far.

The *GHT* genes from *S. pombe* not only encode glucose transporters (*GHT1*, *2* and *5*) but also gluconate transporters (*GHT3* and *4*). *GHT2* and *5* are not repressed by glucose, in contrast to *GHT1*, *GHT3* and *4*. Nevertheless, *GHT5* is expected to be a high affinity glucose transporter, but so far no promoter studies about any of the *GHT* gene group of fission yeast is available [50].

Expression of another set of hexose transporters–the *HGT* genes–was studied in *Candida albicans*. In conjunction with derepressed genes (and promoters) *HGT9*, *HGT10*, *HGT12* and *HGT17* are most interesting for this review, since they are strongly induced at low glucose concentrations (0.2% w/v) [31].

Not surprisingly, also hexose transporters in the industrial workhorse *Pichia pastoris* attracted interest in the context of natural promoters and strain engineering aiming at methanol-free alcohol oxidase (*AOX1*)-promoter controlled expression. The only two known hexose transporters are *Pp*Hxt1 and *Pp*Hxt2. *Pp*Hxt1 is related to the *S. cerevisiae HXT* genes, is induced at high glucose concentrations and seems to play a minor role in *P. pastoris. Pp*Hxt2 is more species specific, has characteristics of a high-affinity glucose transporter, but is also responsible for main glucose transport during high glucose concentrations. Interestingly, a deletion of *Pp*HXT1 leads to a hexose mediated induction of P_AOX1_[14], most probably due to the resulting low intracellular glucose concentration in such deletion variants.

Additionally, Prielhofer and colleagues described the use of several *Pichia* species’ hexose transporters as new promoter targets with green fluorescent protein (GFP) as reporter and, therefore, provided a potential alternative to methanol induced promoters [40] or engineered synthetic promoters, which also do not need methanol for induction [33].

### SUC2 promoter

The *SUC2* gene of *S. cerevisiae* encodes an invertase (beta-fructofuranosidase) and is inducible by sucrose. As for other glucose repressed genes, also the promoter of *SUC2* enables expression to a high level without any external inducer. Similarly to *HXT* genes, derepression of *SUC2* promoter takes place when the level of glucose (or fructose as well) is decreasing below a certain level (0.1% w/v); *SUC2* promoter, interestingly, gets repressed again when glucose concentration drops to zero. In cultivations with glycerol as only (non-repressing) carbon source, the expression of *SUC2* was shown to be 8-fold lower than expression in media with low glucose concentration [51]. The regulation of the *SUC2*-promoter is subjected to Mig1 and Mig2 binding sites on one hand (repression at high glucose level, [52]) and to Rgt1 repressor on the other hand (repression at lack of glucose, basal *SUC2* transcription). At low glucose concentrations, Mig1/2, as well as Rgt1, are phosphorylated by the Snf1/Snf4 complex and thus transcription of *SUC2* is initiated [53]. Additionally, the promoter activity can be further enhanced by sucrose induction but this is not essential for good promoter activity [51].

P_*SUC2*_ is a very suitable promoter for heterologous protein expression in yeast, and processes have been optimized for several applications, also above laboratory scale. For example, significant results for α-amylase expression by P_*SUC2*_ have been obtained using lactic acid as carbon source, a substrate supporting recombinant gene expression as well as cell growth by providing a fast way of energy production (lactate is converted to pyruvate and enters the TCA cycle). The advantage of an extended cell growth phase driven by a non repressing carbon source opened the possibility for the use of P_*SUC2*_ also in large scale applications [54].

In analogy, *inv1* from *Schizosaccharomyces pombe* was subject of the development of a regulated expression system in *S. pombe*, since also the P_*inv1*_ is repressed by glucose (Scr1 mediated, which is another DNA binding protein recognizing GC-rich motifs within the promoter) and is further inducible by sucrose [55].

*In Kluyveromyces marxianus*, *INU1*, which is a closely related gene to *SUC2* and encodes an inulase enzyme, responsible for fructose hydrolyzation, also carries two putative Mig1-recognition sites [18]. The promoter is activated by addition of sucrose or inulin, the derepression is controlled in a similar way to *SUC2*[56]. P_*INU1*_ was applied to several protein synthesis approaches in *K. marxianus* and *S. cerevisiae*, such as expression of inulase (inuE) or glucose oxidase (GOX) from *Aspergillus niger*[57,58].

### JEN1 promoter

*JEN1* encodes a transporter for carboxylic acids (e.g. lactate, pyruvate) in *S. cerevisiae. JEN1* expression is repressed by glucose and derepressed when glucose level falls below 0.3 % (w/v), reaching a peak of activity at 0.1 % (w/v) glucose. Additionally, a weak P_*JEN1*_ activation by lactic acid was observed, using GFP as reporter gene [59].

The regulation of P_*JEN1*_ by the transcription factor Adr1 and the alternative carbon source responsive activator Cat8 was confirmed [60]. Two Mig1 binding sites in the upstream sequence of *JEN1* were identified [32,61]. Subsequently, however, Andrade and colleagues published an alternative mechanism of regulation, proposing that Jen1 is post-transcriptionally regulated by mRNA degradation, rather than by Mig1 mediated repression [62].

*JEN1* promoter has been successfully applied to Flo1 expression, a protein involved in flocculation processes [59].

### ADH2 promoter

A very popular promoter, used in several yeasts, is the promoter of the alcohol dehydrogenase II gene from *S. cerevisiae*[63]. In contrast to the widely used constitutive yeast ADH1 promoter, P_*ADH2*_ is strongly repressed in presence of glucose, and derepressed as soon as the transcription factor Adr1 binds to the upstream activating sequence UAS1 of P_*ADH2*_. Adr1 is dephosphorylated when glucose is depleting, and the cell switches to growth on ethanol (Adr1 dephosphorylation appears to be Snf1-dependent). There is also a second glucose dependent UAS (namely UAS2), less characterized but likely activated by Cat8 in a synergistic way with Adr1, and thus identified as a CSRE sequence (carbon source responsive element) [24,64]. Furthermore, some other protein kinases, such as Sch9, Tpk1 and Ccr1, that also derepress P_*ADH2*_, influence the expression level of ADH2. Interestingly, there is no typical Mig1-binding site in the *ADH2* promoter sequence; glucose repression is mainly mediated by the Glc7/Reg1 complex [11].

The potential of P_*ADH2*_ was evaluated and compared to the inducible *S. cerevisiae* promoters P_*CUP1*_ and P_*GAL1*_ and turned out to yield the highest level of expression after 48 hours [65].

*S. cerevisiae ADH2* promoter is not the only alcohol dehydrogenase promoter used in expression studies. P_*adh*_ from *S. pombe*, (adh shows high homology with *S. cerevisiae* Adh2 at the protein level) is a frequently used promoter in fission yeast, but is described as being constitutively expressed [66].

The related *ADH4* gene from *K. lactis* is characterized by a strong ethanol induction, and is therefore separately described in Section Induction by non-sugar carbon sources.

A *Pichia*-specific *ADH2* promoter was isolated from *Pichia stipitis* and is–in contrast to *ScADH2*–not glucose- but oxygen-dependent (induction at low O_2_ level). This *PsADH2* promoter was used in the heterologous host *Pichia pastoris* for the expression of *Vitreoscilla* hemoglobin (VHb) [67].

### HXK1, GLK1 promoter

Hexokinase (*HXK1*) and Glucokinase (*GLK1*) in *S. cerevisiae* are involved in the first reaction of glycolysis, the phosphorylation of glucose, and are activated when the cell is entering a starvation phase or when switched to another carbon source [36]. Both enzymes are not expressed in presence of high glucose levels (subjected by a classical Mig1 repression; [10]), but become derepressed as soon as glucose is depleting. In case of *GLK1*, a 6-fold increase of expression level by derepression and further 25-fold induction by ethanol was reported [35]. *HXK1*, in comparison, is 10-fold repressed by glucose in dependence of Hxk2 protein [36] and was listed as one of Thierfelder’s glucose dependent promoters with average strength, when it is induced at low glucose concentration [39].

P_*HXK1*_, for instance, was successfully applied to the expression of a *GST*-cry11A fusion protein in *S. cerevisiae*[68] or, in more recent years, to the expression of bovine β-casein [69]. In case of P_*GLK1*_, no application in terms of recombinant protein production was reported.

### Carbon source dependent inducible promoters

Other promoters are derepressed in absence of glucose and additionally need to be induced by an alternative carbon source to obtain full expression efficiency (Table 4). The inducer is either produced by the cell in course of time or has to be provided in the medium.

**Table 4:** Yeast promoters induced in dependence of carbon sources and their regulator elements

**Promoter**	**Protein function**	**Organism**	**Induced by (strength)**	**Repressed by**	**Regulating sequence**	**DNA-bindingtarget protein**	**Ref.**
GAL1	Galactose metabolism	*S. cerevisiae*	Galactose (1000×)	Glucose	-390 to -255	Gal4	[70]
					-201 to -187	Mig1	[71]
GAL7	Galactose metabolism	*S. cerevisiae*	Galactose (1000×)	Glucose	-264 to -161	Gal4	[70]
		*K. lactis*	Galactose		No information available		
GAL10	Galactose metabolism	*S. cerevisiae*	Galactose (1000×)	Glucose	-324 to -216	Gal4	[70]
	Galactose metabolism	*C. maltosa*	Galactose	Glucose	No information available		
PIS1	Phosphoinositol synthase	*S. cerevisiae*	Galactose, hypoxia (2×), zinc depletion (2×)	(glycerol)	-149 to -138	Rox1	[72]
						Gcr1	
					-224 to -205	Ste12	
						Pho2	
					-184 to -149	Mcm1 (2×)	
LAC4	Lactose metabolism	*K. lactis*	Lactose, galactose (100×)	-	-173, -235	RNA-Pol II	[73]
					-437 to -420	Lac9	
					-673 to -656		
					-1088 to -1072		
MAL1	Maltase	*H. polymorpha*	Maltose sucrose	Glucose	No information available		[74]
MAL62	Maltase	*S. cerevisiae*	Maltose sucrose	Glucose	-759 to -743	Mal63	[75][11]
AGT1	Alpha-glucoside transporter	Brewing strains *S. cerevisiae*, *S. pastorianus*	Maltose sucrose	Glucose	Divergent (strain dependent)	Mig1	[76]
						Malx3	
ICL1	Isocitrat lyase	*P. pastoris*	Ethanol (200×)	Glucose	No information available		[77]
		*C. tropicalis*	Ethanol	Glucose	No information available		[78]
		*S. cerevisiae*	Ethanol (200×)	Glucose	-397 to -388	Cat8, Sip4	[21][79]
					-261 to -242	URS	[79]
					-96	RNA-Pol II	[77]
FBP1	Fructose-1,6- bisphosphatase	*S. cerevisiae*	Glycerol, acetate, ethanol (10×)	Glucose	-248 to -231	Hap2/3/4 (2×)	[80]
					No information available	Cat8, Sip4	[21]
PCK1	PEP carboxykinase	*S. cerevisiae*	Glycerol, acetate, ethanol (10×)	Glucose	-480 to -438	Cat8, Sip4	[21][80]
	PEP carboxykinase	*C. albicans*	Succinate, casaminoacids	Glucose	-320 to -123	Hap2/3/4 (2×)	[80]
					-444 to -108	Mig1 (3×)	[80]
GUT1	Glycerol kinase	*S. cerevisiae*	Glycerol, acetate, ethanol, oleate	Glucose	-221 to -189	Adr1	[81]
					-319 to -309	Ino2/4	[81]
CYC1	Cytochrome c	*S. cerevisiae*	O_2_ (200×), lactate (5-10×)	Glucose	No information available		[82]
ADH4	Alcohol dehyrogenase	*K. lactis*	Ethanol	-	-953 to -741	UAS	[83]
AOX1, 2	Alcohol oxidase	*P. pastoris*	Methanol	Glucose	-414 to -171	Mxr1	[84][85]
AUG1, 2	Alcohol oxidase	*P. methanolica*	Methanol	Glucose	No information available		[84]
DAS1	Dihydroxy- acetone- synthase	*P. pastoris*	Methanol	Glucose	-980 to -1	Mxr1	[84]
FDH	Formate dehydrogenase	*H. polymorpha*	Methanol	Glucose	No information available		[84]
FLD1	Formaldehyde dehydrogenase	*P. pastoris*	Methanol, methylamine, choline	Glucose	No information available		[84]
POX2	Peroxisomal protein	*Y. lipolytica*	Oleate	Glucose	No information available		[86]
PEX8	Peroxisomal protein	*P. pastoris*	Oleate methanol (3-5×)	Glucose	-1000 to -1	Mxr1	[87][88]
INU1	Inulase	*K. marxianus*	Fructose, Inulin, Sucrose	Glucose	-271 to -266	RNA-Pol II	[56]
					-163 to -153	Mig1	[58]

Galactose, maltose, sucrose, and some other fermentable carbon sources, as well as oleate, glycerol, acetate or ethanol, as non-fermentable carbon sources, can be considered as alternative inducers for regulated gene expression, since the genes that are involved in the particular metabolism are repressed, as long as the preferred carbon source glucose is available.

### Induction by carbohydrates

#### Induction by galactose

The promoters of the *S. cerevisiae GAL* genes are the most typical and most characterized examples of galactose-inducible promoters. They are strongly regulated by *cis*-acting elements, depending on glucose level, whereupon galactose is acting as the main inducer [70].

**Figure 3:**
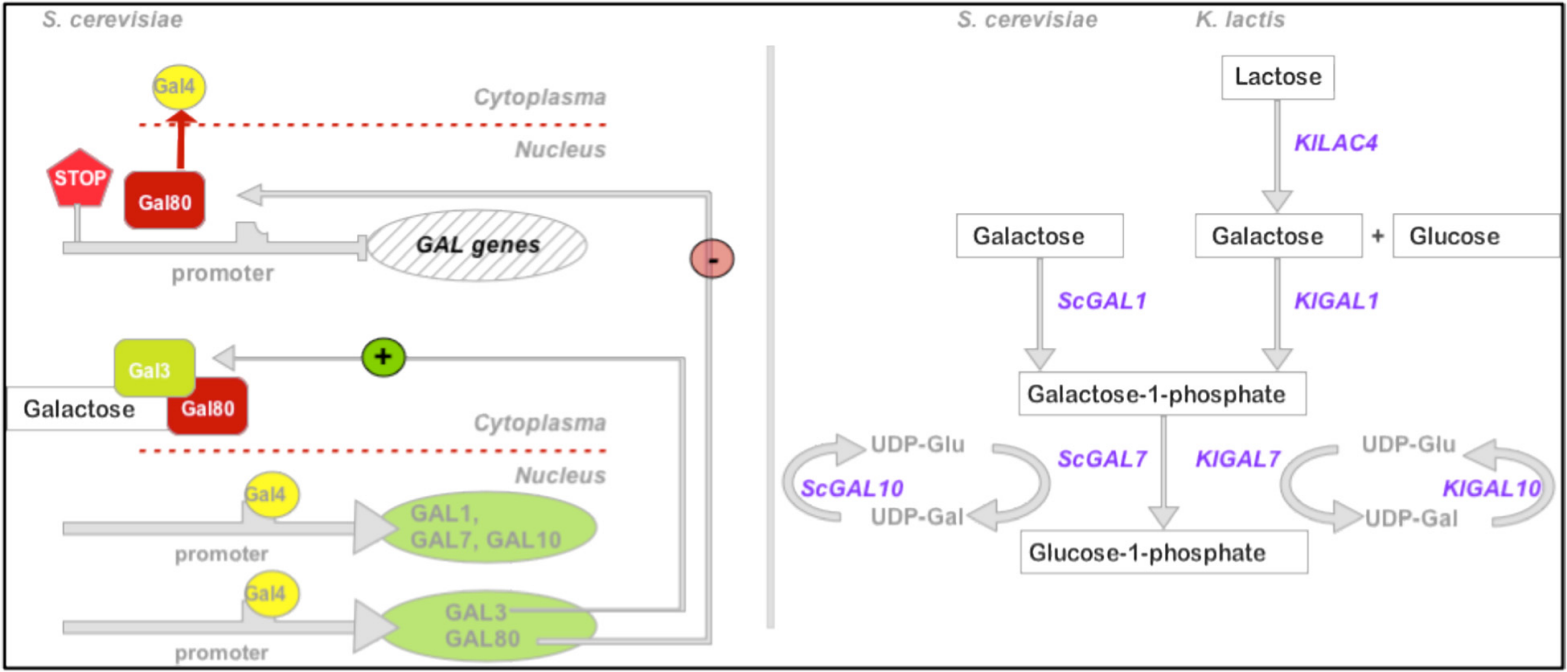
Regulation and function of the GAL genes.

Gal6 and Gal80 are negative regulators of Gal4, which is classified as the activator of the main proteins of galactose utilization pathway *GAL1* (galactokinase), *GAL7* (α-d-galactose-1- phosphate uridyltransferase) and *GAL10* (uridine diphosphoglucose 4-epimerase) [89], as shown in Figure 3. Negative regulators for *GAL* genes have been shown to work in synergy with Mig1 [71]. Gal3 is expected to act as a signal transducer that forms a complex with galactose and Gal80, further releasing Gal4 inside the nucleus and activating *GAL1*, *7 and 10* expression [90,91].

P_*GAL1*_ and P_*GAL10*_ are widely used in *S. cerevisiae* for recombinant protein production, for which different cultivation protocols have been developed. The crucial point is the maintenance of a low glucose level, which is important for efficient induction [92]. Since also galactose concentration decreases during activation of the galactose utilizing pathway, the inducing effect diminishes over time. The high cost of galactose feeding demands a strategy to overcome this problem [93,94]. Several authors have generated *Saccharomyces cerevisiae* gal1 mutant strains that lack the ability to use galactose as a carbon source. Furthermore, *MIG1* and *HXK2* were disrupted to circumvent glucose repression [91,92,94]. Consequently, P_*GAL10*_ is induced even at low galactose concentrations, while presence of glucose does not affect promoter activity. In this case, the optimum concentration of galactose for induction was reported to be 0.05% (w/v) for expressing human serum albumin [92]. Interestingly, Ahn and colleagues found out that P_*GAL10*_ works under anaerobic conditions as well, and easily keeps up with other promoters (P_*PGK*_, P_*PDC*_ or P_*ADH1*_) for fermentative application. Therefore, P_*GAL10*_ is another strong promoter suitable, for example, for microaerobic or anaerobic processes like bioethanol production [95].

Other yeast genera than *Saccharomyces*, like *Kluyveromyces* or *Candida*, present homologous protein functions for galactose utilization. In *K. lactis*, Lac9 resembles the function of Gal4 and is blocked by *Kl*Gal80, which is very similar to Gal80 from *S. cerevisiae*. In contrast to *S. cerevisiae*, there is no Gal3 equivalent in *K. lactis*. Galactose metabolism is mediated by *Kl*Gal1, *Kl*Gal7 and *Kl*Gal10 [11][96]. Besides, *Kl*Lac9 additionally activates *Kl*Lac4, a β-galactosidase, responsible for lactose-utilization (see also Section Induction by lactose). In contrast to *S. cerevisiae*, the regulatory genes of the *K. lactis GAL* expression are not strongly repressed by glucose.

Gonzalez and colleagues took advantage of the resembling *lac-gal*-regulon in *K. lactis* and applied the *S. cerevisiae* promoter P_*GAL1*_ to express *Trigonopsis variabilis* D-aminoacid oxidase (*DAO1*) in *K. lactis*[97].

*GAL1* and *GAL10* promoters of *Candida maltosa* have been successfully isolated, with the intention to create a functional expression system in this species, and were tested with *K. lactis LAC4* as a reporter gene. Both promoters were applied to high level expression of several cytochrome P450s, encoded by the *ALK* gene cluster. P_*GAL1*_ and P_*GAL10*_ of *C. maltosa* were integrated into a low-copy and a high-copy plasmid, respectively, and CO-spectra were measured to prove the P450 expression. In the low-copy plasmid the authors obtained an expression level of 0.96–1.21 nmol/mg wet cell weight, whereas quite notably the high-copy plasmid enabled a 3-fold amount of expressed protein [98]. Other important expression hosts, such as *P. pastoris*, lack a functional pathway and the respective promoters for galactose metabolism.

*PIS1* (phosphatidylinositol-synthase, protein involved in the synthesis of phospholipids) presents an unusual behavior, since it is not subjected to conventional glucose repression in *S. cerevisiae*, but is known for increased transcription as soon as galactose is present in the medium. Interestingly, the presence of glycerol leads to a significant decrease of expression, while the expression level was not affected in glucose-containing medium. The regulatory mechanism is mainly mediated by Mcm1, a DNA-binding protein, which further interacts with another modulating protein, Sln1 [99]. Additionally, *PIS1* is repressed at anaerobic conditions [72] and is responsive to zinc (increased *PIS1* expression after zinc depletion was reported; [100]). Hence, *PIS1* provides a range of possibilities for regulated gene expression with one single promoter.

There are no *PIS1* promoter applications reported yet, but Stadlmayr and colleagues described the *Pichia pastoris* P_*PIS1*_ in a comparative *Pichia* promoter study, where the promoter activity was accounted for being rather low, when different carbon sources (glucose, glycerol and methanol) were tested [101,102].

#### Induction by lactose

A distinctive feature of *K. lactis* is the ability to use lactose as a carbon source. Primarily the proteins of lactose and galactose metabolism are co-regulated by the *lac-gal*-regulon. A lactose permease (Lac12) is transporting lactose into the cell, where it is cleaved to glucose and galactose by a β-galactosidase (Lac4). Subsequently, the galactose metabolism is activated, involving genes (*Kl*Gal1, *Kl*Gal7 and *Kl*Gal10) corresponding to the *S. cerevisiae* counterparts described above [103].

The gene products of lactose and galactose metabolisms are controlled by an activator protein Lac9 (= *Kl*Gal4) and all of them are induced by lactose or galactose.

Interestingly, the lactose utilization genes are not repressed by glucose in *K. lactis*, while the galactose metabolism is weakly repressed [11,104]. The low catabolite repression, along with strong induction potential, is one of the advantages of many *K. lactis* promoters.

The promoter P_*LAC4*_ has been successfully used for recombinant protein production in *K. lactis*[105]. Significant applications consisted, for example, in the controlled expression of prochymosin, important in the context of cheese production [106], or in the expression of *Rhizopus oryzae* α-amylase [107]. The consumption of the inducer is a problem in practical applications of P_*LAC4*_ also in this organism: *K. lactis* strains with disrupted *KlGAL1* were generated to prevent an early consumption of the inducer, following a similar strategy as the one showed for *S. cerevisiae*[108,109].

An interesting side effect is also the occurrence of Pribnow box-like sequences in the native promoter, which enables P_*LAC4*_ to constitutively express heterologous proteins in *E. coli*. However, this feature is rather unwelcome for a typical protein expression process, since a prior correct assembling of the constructs in *E. coli* as an intermediate host can be problematic. With the goal to circumvent this inconvenience, a set of P_*LAC4*_-variants, mutated in Pribnow box-like sequence, has been developed. Such a promoter modification allowed to successfully express bovine enterokinase, whose expression had been problematic before [110].

#### Induction by maltose

Maltose utilization is a feature of several yeasts, among which *S. cerevisiae*, *Hansenula polymorpha* (the only methylotrophic species for which this phenotype was reported) and *K. lactis*. The *MAL* gene group is repressed by glucose and induced by maltose and sucrose. There are up to 5 unlinked *MAL* loci in yeast (*MAL1*, *MAL2*, *MAL3*, *MAL4*, *MAL6*), and each of them consists of a permease (*MALx1*), a maltase (*MALx2*) and an activator protein (*MALx3*) [74]. The promoter region for *MALx1* and *MALx2* is a bidirectionally active intergenic region, consisting of an UAS, 2 symmetrically organized TATA-boxes, 2 Mig1 binding sites and intermediate tandem repeats, which are assumed to regulate the expression level of *MALx1* and *MALx2*. This bidirectional promoter was applied to the simultaneous expression of reporter genes *MEL1* and lacZ [111]. Several authors highlighted the potential of *MAL*-promoters in expression vectors for regulated protein synthesis, by using maltose as an inducer [112]. For example, using P_*MAL62*_ from *S. cerevisiae* provided similar expression results as P_*GAL1*_, when LexA was expressed as a reporter gene. Notably, background expression driven by P_*MAL62*_ was definitely higher (compared to P_*GAL1*_) under non-repressing and non-inducing conditions. Nevertheless, the expression of a very toxic protein, cyclin A from *Drosophila* was efficient for the maltose regulated protein synthesis by P_*MAL62*_ compared to its constitutive expression with P_*ADH1*_[113].

*AGT1*, which encodes a α-glucoside transporter, is highly homologous to the *S. cerevisiae MAL61.* P_*AGT1*_ sequence from several beer yeast strains (*S. cerevisiae*, *S. pastorianus*), was recently analyzed. *AGT1* is repressed by glucose in a similar manner in all tested strains (it showed to be Mig1-dependent), while derepression and maltose induction strength are strain-dependent, probably due to a certain divergence in *AGT1* promoter sequences. The regulation of maltose induction is dependent by the *MAL* activator proteins, [76].

In *H. polymorpha*, the native P_*MAL1*_ performed very well–also in comparison to the commonly used P_*MOX*_, especially when the promoter was induced by sucrose. Furthermore, *H. polymorpha* P_*MAL1*_ was transferable to another maltose utilizing yeast species–*S. cerevisiae* -, for recombinant expression of native maltase [75].

### Induction by non-sugar carbon sources

#### Induction and derepression by ethanol, glycerol or acetate

Glycerol is a very relevant inducer of many promoters; interestingly, glycerol is often used to “derepress” a promoter, prior to actively induce transcription activation by another inducer, such as ethanol, methanol or acetate. Most of related genes are involved in gluconeogenesis. Below, the most important promoter sequences belonging to this group will be described.

One meaningful promoter in this category is the promoter of *ICL1*, which encodes for isocitrate lyase, a key enzyme of the TCA and glyoxylate cycle, enabling the cell to grow on non-fermentable carbon sources. It is repressed by glucose, derepressed by depletion of glucose and strongly induced by ethanol or acetate. P_*ICL1*_ is mainly regulated by the two C_6_-zinc finger proteins Cat8 and Sip4 (see Table 1), which bind to a UAS as soon as glucose is depleted and ethanol or acetate are available [79].

*ICL1* promoter sequences from several yeasts such as *S. cerevisiae*, *P. pastoris*, *Yarrowia lipolytica* or *Candida tropicalis* are well established and frequently applied to protein expression [77,78,114]. In *K. lactis*, *ICL1* is assumed to be regulated without a Mig1 repressor, even if the derepression and induction are mediated by the Snf1/Snf4 complex [115]; it is still unclear if other repressor proteins are involved with URS regulation of *ICL1*.

In *S. cerevisiae*, the 5′ upstream region of *ICL* from *Candida tropicalis* is often used as an inducible promoter; its optimum glucose concentration for derepression was measured at 0.5% (w/v), when *Rhizopus oryzae* lipase was expressed [116]. While expressing secreted β-galactosidase, the induction with acetate leads to a 300-fold enhancement of product activity. It needs to be mentioned that this level of expression is protein-dependent (e.g. expression of lipase yielded only a fraction of the protein amount after induction compared to its expression under derepressed promoter condition [116]). Therefore, the volumetric activity of an expressed enzyme does not necessarily correlate with the strength of transcription. This might also be a reason why in *Pichia pastoris* the native P_*ICL1*_ was praised as a good alternative for methanol free protein production [77], while on the other hand, according to a recent review, the transcription levels of this promoter in *Pichia pastoris* appear to be lower than with the classic P_*AOX1*_ or P_*GAP*_[117].

The P_*ICL1*_ of *Y. lipolytica* is a standard promoter used for this host and was reported to be induced about 10-fold by ethanol, when β-galactosidase was expressed [114]. Besides, it has been reported to be inducible by fatty acids and alkanes [118].

A special case is represented by *ALG2* in *H. polymorpha*, which encodes another isocitrate lyase with 50–60% sequence homology to *ICL* of other yeasts. The promoter of *ALG2* is activated by derepression at low glucose level (0.2% w/v) rather than by ethanol induction [37].

The promoter region of *FBP1*, encoding fructose-1, 6-bisphosphatase, was analyzed in several occasions, concerning upstream regulating sequences [80,119]. P_*FBP1*_ is repressed by sugars like glucose, shows a Mig1 binding site in the upstream sequence from -200 to -184 [11] and carries a Cat8 and Sip4 recognition site (UAS2) for activation of transcription when non-fermentable carbon sources (ethanol, acetate, glycerol) are available [21,22]. Additionally, P_*FBP1*_ was reported to have another regulatory sequence (UAS1), showing a different sensitivity to glucose than UAS2 [119], a genetic arrangement unique within this group of presented promoters.

Within the group of budding yeast, no practical applications of P_*FBP1*_ have been found: only the *fbp1*+ promoter from fission yeast was mentioned several times as an opportunity for controlled gene expression in *S. pombe*[120]. However, this might also be explained by the fact that Fbp1 activity in glycolysis is also strongly regulated on the protein level, and not mainly by transcription.

The PEP carboxykinase (*PCK1*) promoter, which is inducible in absence of glucose by glycerol, ethanol, acetate or lactate as well, was already isolated from several yeasts, like *S. cerevisiae*[80] or *C. albicans*: in particular, the P_*CaPCK1*_ gained popularity within *Candida* community. By means of the *S. cerevisiae PCK1* promoter, Cat8 and Sip4 have been identified as responsible activator proteins for transcription as well [21]. It has however to be mentioned, that, at least in the case of *CaPCK1* promoter, other inducers, such as casamino acids or succinate, have been proved to be more efficient regarding expression of *LAC4* in *C. albicans*[121]. This observation was confirmed by an example, where the *CaPCK1* promoter was applied to *Ca*Cse4-expression in *C. albicans* by succinate induction [122] and furthermore by *Ca*Cdc42-expression, which was driven by casamino acid induction [123].

Technically speaking, also the promoters of the gluconeogenetic proteins Acs1 (acetyl-CoA-synthase) or Mls1 (malate synthase) belong to this group, and have been characterized regarding their upstream regulatory sequences [124,125], but to the best of our knowledge they have not been applied for protein production yet.

The *S. cerevisiae* glycerol kinase (*GUT1*) is another example of a gene whose expression is mainly induced by glycerol, but also by ethanol, lactate, acetate or oleate. Complete depletion of glucose is necessary to derepress the promoter. The regulation mechanism is subjected to Adr1 and Ino2/4 activation, and repression by Opi1 activity. Even if there might be a Mig1-binding site, this repressor seems to play a minor role [81]. The use of the *P. pastoris* P_*GUT1*_ promoter was proposed quite recently and was successfully applied to expression of β-lactamase as a model protein [126].

The *CYC1* (cytochrome c) gene product is an important element of the electron transport in *S. cerevisiae* and is repressed under anaerobic conditions and in presence of glucose. The intracellular heme level mediates the O_2_-dependent activation of UAS1 element in the *CYC1* promoter region by binding of Hap1. UAS2 binds the Hap2/3/4/5 complex, and is activated by any non-fermentable carbon source [82]. Induction with O_2_ increases expression about 200-fold, whereas lactate-induction is not as effective (5–10 fold) [127].

In the respiratory yeast *K. lactis*, *CYC1* is expressed to a high level too, but glucose repression is also in this case almost irrelevant because the major part of expression is fulfilled by O_2_-induction and UAS1 activation [128].

Cytochrome c is a highly conserved protein in several eukaryotes, and is therefore easy to transfer between different yeast species. In many *S. cerevisiae* vectors, the terminator of *CYC1* gene is used for termination of transcription. Nonetheless, the promoter region of *CYC1* is not particularly exploited, in any case often evaluated as hybrid promoter with *GAL10* (UAS_G_-GAL10/CYC1). This construct consists of 365 bp of *GAL10*, including a UAS sequence, and the core promoter of *CYC1* (TATA-Box, transcription start site and first four basepairs of *CYC1* gene). Da Silva & Bailey have applied such hybrid promoter, among others, in order to determine the influence of different promoter strengths on fermentative protein expression in yeast, and as a result UAS_G_-GAL10/CYC1 promoter showed moderate strength compared to P_*GAL1*_, when it was induced with galactose [129]. Nevertheless, one example of successful application of the hybrid promoter is the expression of HbsAg and preS2-S in *S. cerevisiae* for HBV vaccine preparation [130].

The use of the *K. lactis ADH4* promoter was patented by Falcone and colleagues [131]. It is located in the mitochondria, is not repressed by glucose but strongly induced by ethanol. The important control region for regulation of ethanol induction was found to be located between -953 and -741 [83].

For the sake of completeness, it has to be mentioned that also the *S. cerevisiae ADH2* promoter is induced by ethanol, but due to its efficient repression/derepression mechanism this promoter was described in Section Promoters derepressed by carbon source depletion. The same applies to the hexokinase genes *HXK1* and *GLK1*.

#### Induction by methanol

This promoter type has been sufficiently reviewed in the past by several authors, and will therefore be mentioned only briefly. For further detailed information, we refer the reader to the corresponding literature (see below).

The use of methanol as an inducer is confined to methylotrophic yeasts, like *Pichia pastoris*, *Pichia methanolica*, *Hansenula polymorpha* or *Candida boidinii*, which are able to metabolize methanol as a carbon source [132]. The most established promoters comprise those from genes encoding alcohol oxidases (namely P_*AOX1*_ and _-*2*_ in *P. pastoris*, P_*AUG1*_ and _-*2*_ in *P. methanolica*, P_*MOX*_ in *H. polymorpha*, P_*AOD1*_ in *C. boidinii*), dihydroxyacetone synthases (P_*DAS1*_ and P_*DAS2*_ in *P. pastoris*, P_*DAS*_ in *H. polymorpha*, P_*DAS1*_ in *C. boidinii*) and formate dehydrogenases (P_*FDH*_ in *H. polymorpha*, P_*FDH1*_ in *C. boidinii*). All of them are elements of the methanol utilization (MUT) pathway, and are repressed by glucose and strongly induced by addition of methanol (importantly, they are also derepressed by a non-fermentable carbon source, e.g. glycerol). Especially *H. polymorpha* P_*MOX*_ shows a significant derepression effect in presence of glycerol, since protein activity is already 80% of the methanol induced status. A special case in this context is the group of formaldehyde dehydrogenases (P_*FLD1*_ in *P. pastoris*, P_*FLD*_ in *P. methanolica*, P_*FLD*_ in *H. polymorpha*), which are not only negatively regulated by glucose, but additionally are responsive to methylamine or choline induction [84,101,133].

At present, a set of engineered promoter variants based on these natural sequences of the MUT pathway genes have been developed. Such modified promoters (e.g. P_*MOX*_ in *H. polymorpha* and P_*AOX1*_ in *P. pastoris*) are no longer methanol inducible, showing in most cases either an inducible phenotype from molecules other than methanol, or a more pronounced derepressed phenotype [134,135].

In case of P_*FLD*_, Resina and colleagues exploited an advantageous characteristic of the promoter (P_*FLD*_ is inducible by methylamine) thereby circumventing methanol induction [136].

*PEX8* is a peroxisomal protein (formerly *PER3*) in *P. pastoris*, whose promoter leads to a moderate expression level on glucose. A weak induction by methanol or oleate (3–5 fold) has been reported [87,118]. The main regulator protein in P_*PEX8*_ is Mxr1, which is characteristic for all methanol inducible genes in *Pichia* and binds the promoter in a 5′-CYCCNY-3′ motif [88]. It remains to be demonstrated if multiple Mxr1 binding sites such as in the P_*DAS*_ and P_*AOX*_ promoters would increase P_*PEX8*_ strength.

P_*PEX8*_ was chosen for instance in the framework of Pex14 characterization, and was applied under methanol- and oleate-inducing conditions, respectively [137].

#### Induction by oleate

Oaf1 and Pip2 are important DNA-binding proteins for the transcriptional activation of oleate responsive proteins in yeast. In many cases (e.g. *CTA1*; *peroxisomal catalase*, *POX1*; *peroxisomal acyl CoA oxidase*, *FOX3*; *3-ketoacyl CoA thiolase*, *PEX1*; *peroxisomal biogenesis factor 1*) also Adr1 is involved in initiating gene transcription [138]. Most of these proteins are functionally connected to the peroxisomes and are mainly involved in β-oxidation. For example *POX1*, *FOX3* (= *POT1*), *ECI1* and *PEX11* are strongly induced by oleate and repressed by glucose, whereupon a significant derepression already occurs in presence of glycerol. Besides, *PEX5*, *CRC1*, *CTA1* and *QDR1* are also induced by oleate, although at a lower level [12].

In terms of industrial applications, no relevant oleate inducible promoters have been reported for *S. cerevisiae* so far. Up to now, especially, *POX2* and *POT1* promoters from *Y. lipolytica*, which are also activated by oleate, have been validated for recombinant protein synthesis of lipase in *Y. lipolytica*[138]. In the meantime P_*POX2*_ has been frequently used, especially, when hydrophobic substrate conditions were required. The performance of P_*POX2*_ was further improved, testing human interferone alpha 2b expression, by co-feeding glucose at a limited rate during induction with oleate [139].

## Conclusions

This review describes the current state of art for a set of potential promoters for controlled protein synthesis, out of several yeasts. Especially in case of inducible promoters, the presented genetic tools are already well established, with several examples now summarized within this work. Nevertheless, also some less popular promoters show interesting features, which might be enhanced by promoter engineering: such a technique, despite its potential, is not yet very common for promoter improvements.

Generally, any gene subjected to derepression at low glucose concentrations, opens up the potential of carrying a strong promoter sequence. Referring to transcriptome analysis covering 31% of the genome [140,141], about 163 genes from *S. cerevisiae* were upregulated at glucose-limited conditions. Many of these genes are still poorly characterized, and their function is not known yet. For instance, *YGR243* promoter from *S. cerevisiae* was already introduced as an interesting promoter tool [39], whereupon P_*YGR243*_ could easily keep up with P_*HXK1*_.

A comprehensive knowledge of promoter elements is also helpful in terms of the development of synthetic promoters, since this field of research is relatively new, but gained increased popularity within the last ten years. Sequences of strong natural promoters are combined, and transcription factor binding sites are deleted or amplified with the objective of obtaining a new, more convenient promoter sequence [142].

Very recently, Blazeck and colleagues presented a set of synthetic yeast promoters by assembling very strong transcriptional enhancing elements (coming from CLB2, CIT1, GAL1, respectively) with the core of a particular promoter. The essential finding was a direct proportion between the number of additional UAS and promoter activity [143]. Interestingly, most yeast promoter studies are still focused on endogenous promoters and rarely on heterologous applications or fully orthogonal systems.

A broad knowledge of different potentials of promoter elements paves the way for creating a comprehensive promoter tool box and facilitates protein synthesis for appropriate applications.

## Competing interests

The authors declare that they have no competing interests.

## Authors’ contributions

KW and AC collected all the relevant publications, arranged the general structure of the review and drafted the text; AC, AG and MW revised and amended the general flow. KW produced tables and figures. All authors read and approved the final manuscript.
